# Associating multiple longitudinal traits with high-dimensional single-nucleotide polymorphism data: application to the Framingham Heart Study

**DOI:** 10.1186/1753-6561-3-s7-s47

**Published:** 2009-12-15

**Authors:** Sandra Waaijenborg, Aeilko H Zwinderman

**Affiliations:** 1 Department of Clinical Epidemiology, Biostatistics and Bioinformatics, Academic Medical Center, Amsterdam, PO Box 22700, 1100 DE, The Netherlands

## Abstract

Cardiovascular diseases are associated with combinations of phenotypic traits, which are in turn caused by a combination of environmental and genetic factors. Because of the diversity of pathways that may lead to cardiovascular diseases, we examined the so-called intermediate phenotypes, which are often repeatedly measured. We developed a penalized nonlinear canonical correlation analysis to associate multiple repeatedly measured traits with high-dimensional single-nucleotide polymorphism data.

## Background

Cardiovascular diseases (CVD) are associated with combinations of phenotypical traits, such as increased blood pressure, blood glucose, or cholesterol, and many other risk factors. These traits are in their turn caused by a combination of environmental and genetic factors. Because of the high diversity of pathways that may lead to CVD, our focus lies on the so-called intermediate phenotypes that often have a much stronger relationship with genetic markers. The disadvantage of this approach is that there are many such intermediate phenotypes, and moreover they are often repeatedly measured in patients, e.g., lipid profiles, blood pressure, and glucose. We developed a new method to associate multiple repeatedly measured phenotypical traits with high-dimensional single-nucleotide polymorphism (SNP) data, and illustrate its use with the data sampled in the Framingham Heart Study, as provided by the Genetic Analysis Workshop 16 (GAW16).

We have previously shown that penalized canonical correlation analysis (CCA) can be a valuable tool to study the association between two high-dimensional sets of variables [1,2]. It penalizes the two datasets such that it finds a linear combination of a selection of variables in one set that maximally correlates with a linear combination of a selection of variables in the other set, thereby making the results more interpretable.

Although CCA accounts for the correlation between variables within the same set, it neglects the longitudinal nature of the variables. Furthermore, it does not deal well with categorical data, such as we encounter when dealing with SNP data. We adapted CCA such that it captures the correlation between the multivariate longitudinal responses instead of the correlation between separate measurements within one set. The set with SNP variables is transformed via optimal scaling [3], such that each SNP variable is transformed into one continuous variable, which captures the measurement characteristics of the SNP. Hereafter, this set is penalized such that the number of suspected SNPs is reduced to an interpretable number. Using these approaches, we are able to extract groups of SNPs that have a high association with multiple longitudinal traits.

## Methods

### Data

Data from the Framingham Heart Study containing information about common characteristics that contribute to CVD, together with genetic data of about 550,000 SNPs were provided by the GAW16. Three generations were followed over a period of several years, when at regular time-points characteristics of CVD were measured.

In our analysis we focused on individuals of the offspring generation because the repeated measurements in these individuals were taken under approximately similar conditions; we considered the measurements of total cholesterol, high-density lipoprotein (HDL) cholesterol, triglycerides, and blood glucose, each measured four times. Furthermore, we analyzed the data of the Affymetrix 50 k chip containing 50,000 SNPs. The offspring generation exists of 2,760 individuals, with at most four measurements taken every 7 to 12 years. We only took the individuals (over the age of 17) for whom both phenotypical data and genetic data were available (2,584), and discarded the individuals with more than 5% missing SNP data (6) and individuals who attended only one exam (26). Six individuals with extremely high measures for one of the four traits were also deleted from further analysis.

Monomorphic SNPs and SNPs with a missing percentage of 5% or more were deleted from further analysis. Remaining missing data was randomly imputed once based only on the marginal distribution of the SNP in all other individuals; no assumptions were made about the variation in the imputation. We were primarily interested in common SNP variants, so we grouped SNP classes with less than 1% observations with a neighboring SNP-class; i.e., we grouped homozygotes of the rare allele together with the heterozygotes, if there were less than 1% homozygotes.

This research was in accordance with the Helsinki Declaration of Human Rights, compliant with the data use agreement for the Framingham Heart Study, and was approved by the local medical ethics committee of the Academic Medical Center Amsterdam (date of decision, 1 April 2008).

### Penalized nonlinear CCA

Consider the *n *× *p *matrix **Y**, containing *p *(measured trait) variables, and the *n *× *q *matrix **X **containing the *q *(SNP) variables, obtained from *n *subjects. CCA captures the common features in the different sets by finding a weighted linear combination of all the variables in one set that correlates maximally with a weighted linear combination of all the variables in the other set. These linear combinations are the so-called canonical variates *ω *and *ξ*, such that *ω *= **Y***u *and *ξ *= **X***v*, with weight vectors *u*^*T *^= (*u*_1_, ..., *u*_*p*_) and *v*^*T *^= (*v*_1_, ..., *v*_*q*_)

**Figure 1:**
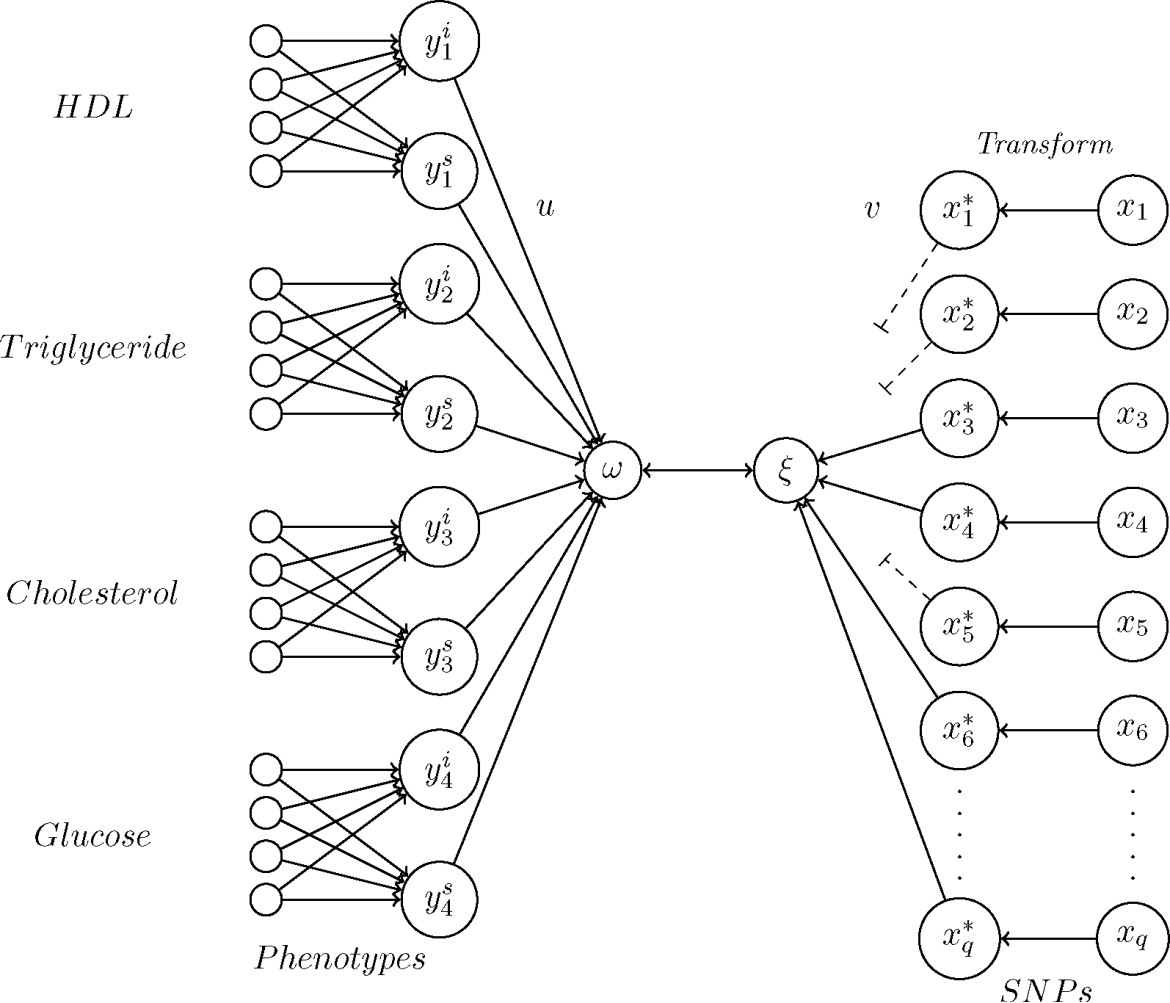
**Penalized nonlinear canonical correlation analysis**. Association between repeatedly measured phenotypes and a large number of SNPs. The longitudinal measured phenotypes are summarized into two measures, one representing the intercept **Y**^**i **^and one the slope (**Y**^**s**^). Each SNP variable (**X**) is transformed into one continuous variables (X*). Hereafter penalized canonical correlation analysis is performed, and only SNPs that contribute to the association are selected.

Because CCA neglects the longitudinal nature of the variables, each repeatedly measured trait is summarized into two measures, one representing the slope and one the intercept for each individual (see next section). Moreover, CCA cannot deal with categorical variables, therefore each SNP variable is transformed into one continuous variable via optimal scaling [3]. Each of the transformed variables are restricted to the measurement characteristics of the SNP. That is,

where *a *is wildtype, *b *is heterozygous, and *c *is homozygous. This restriction indicates that the effect of the heterozygous form of SNP *j *always lies between the effect of the wildtype and homozygous genotype. To make the results more interpretable, the SNP set is penalized using univariate soft-thresholding. The canonical variates are optimized via the following alternating least squares algorithm (see Figure 1):

1. Standardize **Y **and **X**.

2. Set k ← 0.

3. Assign arbitrary starting value ***ω***^1^.

4. Estimate *ω*, *ξ*, *v *and *u *iteratively as follows

Repeat

(a) Obtain the transformed matrix X* by minimizing the distance between  and **X**. That is, *j *= 1,2, ..., *q*, with **G**_j _the *n *× *g*_*j *_indicator matrix for variable *j *with *g*_*j *_the number of categories of variable *j*. Restrict  to obtain . Then . Standardize X*.

(b) Compute  with univariate soft-thresholding, *j *= 1,2, ..., *q *(with *f*_+ _= *f *if *f *> 0 and *f*_+ _= 0 if *f *≤ 0.) and normalize .

(c) k ← k + 1

(d) 

(e) Compute  with linear regression,  and normalize .

(f) 

until  and  have converged.

### Summary method for the repeatedly measured phenotypes

The four traits (total cholesterol, HDL cholesterol, triglycerides, and blood glucose levels) are log-transformed. Because of the linear nature of the repeated measures, for each subject *i *its repeatedly measured trait variables can be summarized into two measures, an intercept (*β*_0_) and a slope (*β*_1_); after correcting for the treatment effect of cholesterol-lowering drugs, according to the following model, at age *t*: *y*_*it *_= *β*_0*i *_+ *β*_*li*_*Age *+ *β*_2*i*_*trt *+ *β*_3*i*_trt* *Age *+ *e*_*it*_, where *y *is the trait and *trt *= 0 if no treatment was taken and *trt *= 1 if cholesterol treatment was taken. Hereafter, the sex-effect was removed from the eight newly obtained intercept and slope variables. The new dataset **Y **contains eight variables, two for each of the four phenotypical measures.

### Model optimization

Optimization of the penalty parameter is determined via *k*-fold cross-validation. The weight vectors *u *and *v *and the transformation functions *τ*_*j *_per SNP variable are estimated for different penalties in the training set and validated in the validation set.

Instead of determining the penalty *λ*, for interpretation it is easier to determine the optimal number of SNP variables. The optimal number of variables are obtained when the mean difference between the canonical correlation of the training and the validation set is minimized, i.e.,

with  and  the weight vectors estimated for the training sets  and  in which subset *j *was deleted and the validation set  transformed following the transformations of the training set .

## Results and discussion

To decrease computation time, the number of SNP variables was reduced, using univariate analysis. Each of the four intercept variables were separately associated with the SNPs set using the optimal scaling method. Only the intercept variables were used because the absolute correlation between the intercept and slope of the same phenotype was at least 0.89. For each intercept variable, the top 10% of SNPs with the highest weights were selected for further analysis, i.e., SNP variables that received a high weight for any of the four intercept variables were selected. This resulted in a data set with 12,682 SNP variables.

**Figure 2:**
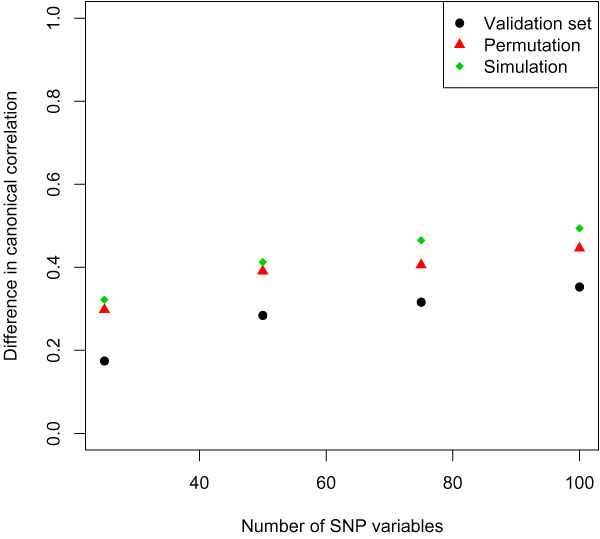
**Determination of the optimal number of SNP variables**.

Next we performed penalized nonlinear CCA; ten-fold cross-validation was performed to determine the optimal number of SNP variables. The optimal number of variables was the number where the canonical correlation of the validation set was closest to the canonical correlation of the training set. Figure 2 shows the effect of the number of selected SNP variables on the difference in canonical correlation of the validation set and the training set. It shows that as the number of selected variables increased, the difference in canonical correlation also increased, which caused the predictive performance of the selected variables to decrease.

If the number of variables is very large, then there is a high probability that a random pair of variables has a very high correlation by chance. To identify a canonical correlation that was large by chance only, a permutation-analysis on the validation sets was performed. We permuted the canonical variate *ξ *(SNP variables) and kept the canonical variate *ω *(phenotypical variables) fixed, then the difference in canonical correlation of the permuted validation sets and the training set was determined (Figure 2).

Furthermore, we performed an additional simulation test in which we associated the actual set of phenotypical variables with a set of permuted SNP variables. For each SNP variable the observations were randomly distributed over the different subjects, then a ten-fold cross-validation was performed (see Figure 2).

The difference in canonical correlation obtained from the permuted data and the simulated data were small (Figure 2), while the difference in the canonical correlation of the real data was smaller, indicating that the simulation set only contained noise data and the results of the real data were larger than would be expected by chance. Figure 2 shows a decreasing trend as the number of SNPs decreases, with a minimum at 25 variables. Although it appears that this trend could decrease even further by minimizing the number of SNPs, we decided not to investigate this to avoid the absence of important SNPs in the final results. We performed penalized nonlinear CCA on the whole dataset, obtaining the 25 SNP variables given in Table 1. The canonical correlation of this model was 0.29.

**Table 1:** Selected SNPs with associating loadings

ID	Chromosome	Position	Gene symbol	Loadings	Cross-loadings
rs4951003	1	203728690		0.2421	0.098
rs12402938	1	207845978	*CAMK1G*	0.061	0.0754
rs9729179	1	230240796	*DISC1*	0.1858	0.0936
rs10803210	1	242600084	*C1orf100*	0.0707	0.0781
rs11885449	2	33464415	*LTBP1*	0.1054	0.0831
rs13385681	2	100453326	*NMS*	0.0414	0.0758
rs10176715	2	227898611	*MFF*	0.0347	0.0762
rs9844754	3	136017093	*EPHB1*	0.0614	0.0763
rs17207005	5	85475541		0.1796	0.0929
rs7700813	5	95166015		0.0811	0.0811
rs1570932	6	90066030	*GABRR2*	0.0732	0.0798
rs4723563	7	36723988	*AOAH*	0.1399	0.0833
rs328	8	19864004	*LPL*	0.1609	0.0849
rs7837540	8	57341761		0.0736	0.0792
rs1458118	8	87785399	*CNGB3*	0.0104	0.0718
rs11997551	8	99204613	*POP1*	0.1036	0.0749
rs721917	10	81696304	*SFTPD*	0.0217	0.0783
rs11028690	11	3615868	*ART5*	0.0425	0.0761
rs1943781	11	101740351	*BIRC2*	0.0467	0.0797
rs2024490	12	95823495	*NEDD1*	0.0931	0.0826
rs1008628	14	104793771	*BRF1*	0.0495	0.0836
rs3764261	16	55550825	*(near CETP)*	0.8674	0.1699
rs7237072	18	66934371		0.2573	0.1016
rs17756963	19	15963980	*LOC646610*	0.0506	0.0809
rs8122970	20	19155712	*SLC24a3*	0.0628	0.0833

In Table 1 and 2 the loadings (correlation of each variable and their respective canonical variates) and the cross-loadings (correlation of each variables with their opposite canonical variate) show how the variables were associated with each other. The selected SNPs were highly associated with the HDL intercept (cross-loading: 0.1419), and had a lower association with the other variables, especially the slope variables. SNP rs3764261 highly associated with the phenotypical variables, while all the other SNPs had comparable cross-loadings.

**Table 2:** Phenotypes with associating loadings

Phenotype	Loadings	Cross-loadings
Cholesterol intercept	0.0547	0.0161
Cholesterol slope	-0.0485	-0.0143
HDL intercept	0.4718	0.1389
HDL slope	-0.0736	-0.0217
Triglyceride intercept	-0.2072	-0.061
Triglyceride slope	0.0952	0.028
Glucose intercept	-0.1979	-0.0583
Glucose slope	0.1413	0.0416

## Conclusion

Our penalized nonlinear CCA is able to identify SNPs that are associated with repeatedly measured phenotypical markers. In this study two important SNPs (rs328 and rs3764261) were found that are located close to or in a gene that has been reported to be associated with HDL concentrations [4].

Although we chose to model the repeated measurements with a linear random effects model, more complex and more flexible models can be easily incorporated in our penalized nonlinear CCA. Besides the first pair of canonical variates, different pairs can be obtained using the residual matrices of the preceding canonical variate pairs.

The family structure in this study was ignored. McArdle et al. [5] noticed that ignoring the family structure mainly affects the type I error rate and not the bias of the point estimate. Because we focused on the point estimates, we expect no major changes when family structure is considered.

## List of abbreviations used

CCA: Canonical correlation analysis; CVD: Cardiovascular diseases; GAW16: Genetic Analysis Workshop 16; HDL: High-density lipoprotein; SNP: Single-nucleotide polymorphism.

## Competing interests

The authors declare that they have no competing interests.

## Authors' contributions

Both authors contributed equally to this analysis.
